# Intermediate progenitor cells provide a transition between hematopoietic progenitors and their differentiated descendants

**DOI:** 10.1242/dev.200216

**Published:** 2021-12-17

**Authors:** Carrie M. Spratford, Lauren M. Goins, Fangtao Chi, Juliet R. Girard, Savannah N. Macias, Vivien W. Ho, Utpal Banerjee

**Affiliations:** 1 Department of Molecular, Cell, and Developmental Biology, University of California, Los Angeles, USA; 2 Molecular Biology Institute, University of California, Los Angeles, USA; 3 Eli and Edythe Broad Center of Regenerative Medicine and Stem Cell Research, University of California, Los Angeles, USA; 4 Department of Biological Chemistry, University of California, Los Angeles, USA

**Keywords:** *Drosophila*, Intermediate progenitor, Blood cell development, Crystal cells, Hematopoiesis, Split GAL4

## Abstract

Genetic and genomic analysis in *Drosophila* suggests that hematopoietic progenitors likely transition into terminal fates via intermediate progenitors (IPs) with some characteristics of either, but perhaps maintaining IP-specific markers. In the past, IPs have not been directly visualized and investigated owing to lack of appropriate genetic tools. Here, we report a Split GAL4 construct, *CHIZ-GAL4*, that identifies IPs as cells physically juxtaposed between true progenitors and differentiating hemocytes. IPs are a distinct cell type with a unique cell-cycle profile and they remain multipotent for all blood cell fates. In addition, through their dynamic control of the Notch ligand Serrate, IPs specify the fate of direct neighbors. The Ras pathway controls the number of IP cells and promotes their transition into differentiating cells. This study suggests that it would be useful to characterize such intermediate populations of cells in mammalian hematopoietic systems.

## Abstract (precis)

**Summary:** Intermediate progenitor cells are a distinct population of multipotent cells that contribute to all three differentiated blood cell types in *Drosophila* and act as signaling centers to neighboring cells.

## INTRODUCTION

The transition from a multipotent progenitor into various types of mature, functional cells is a widely studied process in both *Drosophila* and vertebrates. Foundational studies investigating human hematopoiesis revealed the ability of a multipotent hematopoietic stem cell to differentiate into multiple distinct blood cell types (reviewed by Dzierzak and Speck, 2008; Weissman and Shizuru, 2008). The stem/progenitor and differentiated cell populations are identified and further characterized by expression of unique markers (Coffman and Weissman, 1981; Ikuta and Weissman, 1992; Morrison and Weissman, 1994; Muller-Sieburg et al., 1986; Smith et al., 1991; Uchida and Weissman, 1992). However, the intermediary stage between a stem cell and a differentiated cell is often not well-studied because of a lack of developed tools to target this particular population. *Drosophila* provides an ideal model system with a variety of powerful molecular genetic tools available with which to test and define the function of these intermediate-state cells during the process of hematopoiesis.

Blood cells in *Drosophila* are functionally akin to those derived from mammalian myeloid lineages (reviewed by Evans et al., 2003). As in all invertebrates, *Drosophila* lack lymphoid cells that enable adaptive immunity in vertebrates. The *Drosophila* lymph gland (LG) is the primary site of hematopoiesis during larval development and is made up of multiple paired lobes flanking the dorsal vessel, which functions as the heart (Jung et al., 2005; Mandal et al., 2004). The LG lobes disintegrate during pupariation and the dispersed mature blood cells contribute to the hematopoietic repertoire of the pupa and the adult (Dey et al., 2016; Grigorian et al., 2011).

The anteriorly located lobes are the largest and are referred to as primary lobes that follow a stereotypic pattern of differentiation. Several zones consisting of distinct cell populations have been identified in the primary lobe. The medially located medullary zone (MZ) is composed of blood progenitors, whereas the cortical zone (CZ) houses three types of mature blood cells (Jung et al., 2005). A cell population termed the posterior signaling center (PSC) functions as a niche and produces a variety of secreted signaling ligands that promote progenitor maintenance (Benmimoun et al., 2015; Lebestky et al., 2003; Mandal et al., 2007; Mondal et al., 2011; Oyallon et al., 2016). The cells of the PSC are defined by their expression of the homeotic gene *Antennapedia* (*Antp*) (Mandal et al., 2007).

During first and early second instars, the small primary lobes consist of progenitors that express *domeless* (*dome*) (Jung et al., 2005; Krzemien et al., 2007; Mondal et al., 2011). Hemocyte differentiation initiates at mid-second instar and is marked by *Hemolectin* (*Hml*) and *Peroxidasin* (*Pxn*) expression in the developing blood cells (Irving et al., 2005; Jung et al., 2005; Sinenko et al., 2009; Stofanko et al., 2008). Later in the second and third instar larvae, the number of differentiated cells expands, forming a distinct CZ. The progenitors populate the MZ and continue to express *dome*. The three mature blood cell types (plasmatocytes, crystal cells and lamellocytes) occupy the CZ (Evans et al., 2009; Jung et al., 2005; Krzemien et al., 2010; Minakhina and Steward, 2010). Mature plasmatocytes are positively identified by the presence of the P1 antigen encoded by the *Nimrod C1* (*NimC1*) gene (Kurucz et al., 2007). Crystal cells express Lozenge (Lz), Hindsight (Hnt; also known as Peb) and Pro-phenoloxidase (ProPO) proteins (Jung et al., 2005; Lebestky et al., 2000, 2003; Neyen et al., 2015; Terriente-Felix et al., 2013). Lamellocytes are rarely observed in the LG, but when present they are marked by the L1 antigen encoded by *atilla* (Honti et al., 2009; Lanot et al., 2001; Márkus et al., 2005, 2009; Sorrentino et al., 2002).

A small number of cells residing at the juxtaposition of the MZ and CZ express both *dome* and Pxn but lack mature hemocyte markers, P1 and Lz (Krzemien et al., 2010; Sinenko et al., 2009). This observation suggests a role for these cells in the process of transition from a progenitor to a differentiated fate. Collectively, these cells are referred to as intermediate progenitors (IPs) belonging to an intermediate zone (IZ) (Krzemien et al., 2010; Oyallon et al., 2016). However, thus far no reporter, enhancer, antibody or driver exists to specifically identify or genetically alter the intermediate progenitors. For this reason, molecular pathways that regulate maturation of these transitional cells remain unknown. Here, we describe the development of a ‘Split GAL4’ driver that targets IPs and allows us to monitor and investigate this unique set of transitioning cells. We demonstrate that the IPs are a distinct population of cells that can be increased or reduced in number through genetic manipulation. These IZ cells have a distinct mitotic and gene expression profile compared with cells of the MZ and CZ. These cells are multipotent and contribute to all three differentiated blood cell types. Finally, we find that the IPs act as signaling centers to specify a subset of differentiated blood cell types in neighboring cells.

## RESULTS

### Characterization of the intermediate zone cell population of IPs

**Fig. 1.**
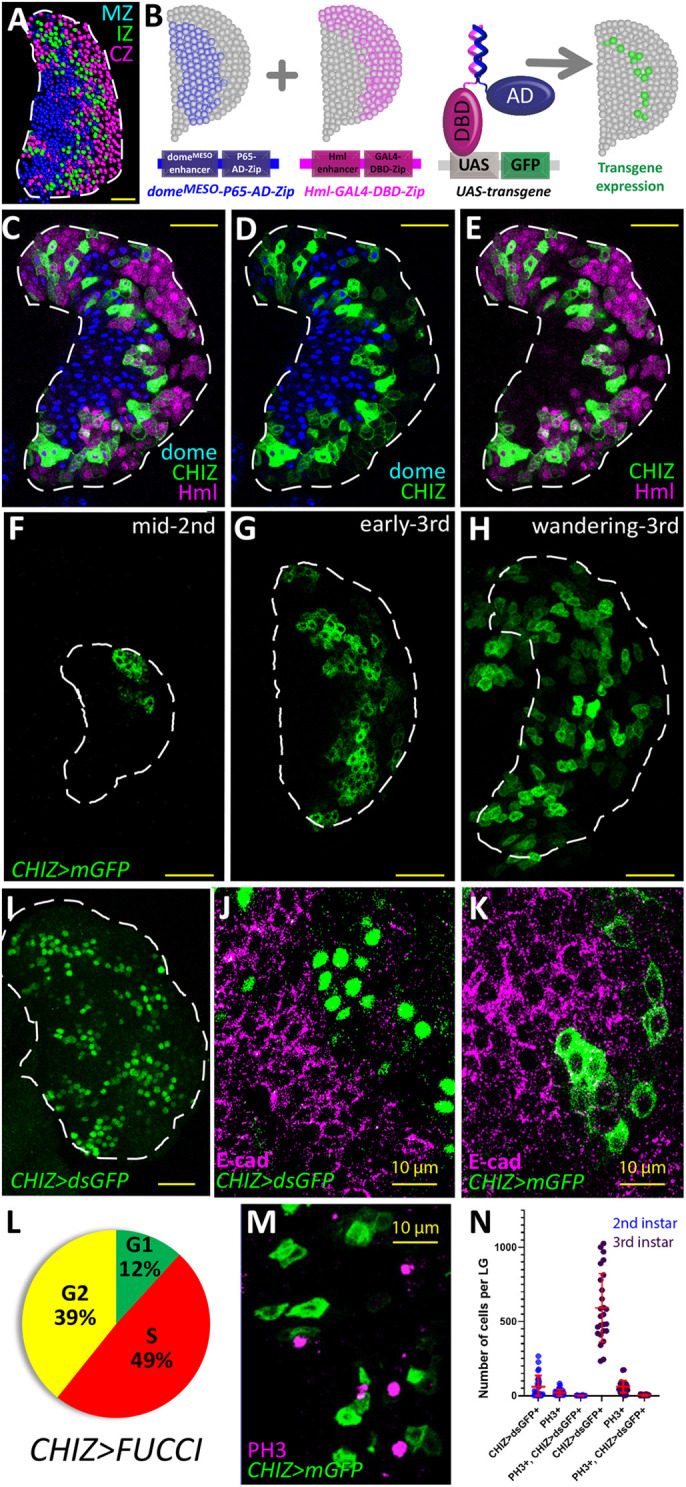
**Characterization of intermediate zone cell population.** (A) Computer rendering of a confocal image of an LG (*dome^MESO^-GFPnls, Hml^Δ^-DsRednls*). Nuclei have been pseudo-colored based on endogenous fluorescence. Progenitors in the MZ are labeled by *dome^MESO^-GFP* and pseudo-colored blue. Differentiated cells in the CZ are labeled by *Hml^Δ^-DsRed* and are pseudo-colored magenta. IZ cells identified by an overlap in expression of both *dome^MESO^-GFP* and *Hml^Δ^-DsRed* are pseudo-colored green. (B) Model depicting the Split GAL4 components used to create *CHIZ-GAL4*. Shown in blue is the expression of a P65 activation domain (AD) in *dome^MESO^*+ cells. Shown in magenta is the DNA binding domain (DBD) of GAL4 which is expressed in *Hml*+ cells. Only the IZ cells with overlapping expression of the AD and DBD express the transgene (GFP, shown in green). (C-E) A third instar LG with fluorescently labeled zones (*dome^MESO^-BFP, Hml^Δ^-DsRed; CHIZ-GAL4, UAS-mGFP*) shows *CHIZ-GAL4* expression (green) juxtaposed between the MZ (*dome*+, blue) and CZ (*Hml*+, magenta). For clarity, for the same LG shown in C, the magenta *Hml* channel is omitted in D, and the blue *dome* channel is omitted in E. (F-H) Developmental progression of *CHIZ-GAL4* expression (*CHIZ-GAL4, UAS-mGFP*). (F) The first appearance of *CHIZ-GAL4* is observed at the distal edge of the mid-second instar LG. (G) During early third instar, *CHIZ-GAL4* expression appears in more cells, but with cells at the periphery lacking *CHIZ-GAL4* expression. (H) In wandering third instar larvae, *CHIZ-GAL4* expression is dispersed throughout the LG. GFP+ cells seen outside of the dashed line belong to the paired primary lobe. (I) IZ marked with nuclear-localized destabilized GFP (*CHIZ>dsGFP*, green). (J) E-cad protein (magenta) present on the progenitor cell membranes ceases its expression within the IZ cells (green). (K) IZ cells (*CHIZ>mGFP*, green) directly abut E-cad+ cells (magenta). (L) Pie chart representing the average percent of *CHIZ-GAL4*-expressing cells in primary LG lobes that are in G1 (green), S phase (red) and G2/early M phase (yellow) as assessed by the expression of the Fly FUCCI indicator. M phase cannot be separately assessed using Fly FUCCI. *n*=115 LGs. (M) Lack of colocalization of *CHIZ* cells (green) with mitotic marker phospho-histone H3 (magenta). (N) Data from second and third instar *CHIZ>dsGFP* LGs stained with PH3 show lack of overlap between IPs and PH3+ cells. *n*=7 LGs for second instar larvae, *n*=13 LGs for third instar larvae. Images in C-E,J,K and M are a single slice of a *z*-stack image. Images in F-I are a maximum intensity projection of the middle third of a *z* -stack of the LG. White dashed lines indicate the edges of the LG primary lobe in A,C-I as discerned from nuclear staining (not shown). Scale bars: 25 μm (A,C-I); 10 μm (J,K,M).

Using a combination of direct drivers of *domeless* (*dome^MESO^-GFP*) and *Hemolectin* (*Hml^Δ^-DsRed*), the IZ cells were seen as an overlapping population at the site of juxtaposition between the MZ and CZ (Fig. 1A). In an effort to positively label and manipulate genetic pathways within these intermediate progenitors, we designed a new ‘Split GAL4’ driver [originally developed by Luan et al. (2006) and refined by Pfeiffer et al. (2008, 2010)] to target the cells with overlapping expression of *dome^MESO^-GFP* and *Hml^Δ^-DsRed* (Fig. 1B). In these constructs, the *dome^MESO^* enhancer is fused to the p65 activation domain and the *Hml^Δ^* enhancer is used to drive the GAL4 DNA binding domain such that only cells that simultaneously express *dome* and *Hml* drive transgene expression downstream of upstream activation sequence (UAS) binding sites. For reasons of brevity, we refer to this driver as *CHIZ-GAL4* (combined hematopoietic intermediate zone*-GAL4*). *CHIZ-GAL4* is the first identified positive marker for the IZ that reliably labels cells in transition from a progenitor to a mature hemocyte. In this paper we use the terms IPs, IZ cells and *CHIZ* cells interchangeably. In principle, other CZ markers, for example *Pxn*, could be used instead of *Hml* to construct a similar driver that marks IPs.

*CHIZ-GAL4* efficiently marked the IPs when used in conjunction with a short-lived fluorophore such as membrane-GFP (mGFP; Fig. 1C-H), Fly fluorescent ubiquitination cell cycle indicator (FUCCI) (Fig. S1E) or a rapidly degrading form of GFP (*dsGFP*; Li et al., 1998; Wang et al., 2012) (Fig. 1I). In an LG fluorescently marked for MZ and CZ cells, *CHIZ>mGFP* (*CHIZ-GAL4; UAS-mGFP*) faithfully labeled cells that express both *dome* and *Hml* and lie at the juxtaposition of the MZ and CZ at all stages of development analyzed (Fig. 1C-E; Fig. S1A-D). Imaging and flow cytometry data show that the IZ comprises 11% (±4.7%, *n*=115 LGs) of the combined number of cells in the two primary lobes of a wandering third instar LG (Fig. S1E,F). Long-lived fluorophores such as eGFP are not useful to specifically visualize the transitioning IZ population owing to their extended perdurance when driven by *CHIZ-GAL4* (Fig. S1G).

*CHIZ>mGFP* expression initiated in a small number of cells at the periphery of the LG at mid-second instar (Fig. 1F). This timing is also coincident with the onset of differentiation. As larval development progressed into the late second and early third instars, IPs increased in number and intensity to form a band of cells in the middle of the LG (Fig. 1G). At the wandering third instar, IPs appeared to be scattered throughout the LG and were notably present in more medial regions compared with earlier stages of development (Fig. 1H). E-cadherin (E-cad), which is required for proper progenitor maintenance, was prominently expressed in the MZ cells (Gao et al., 2013, 2014; Jung et al., 2005) but its expression ceased immediately before the initiation of *CHIZ-GAL4* (Fig. 1J,K). These data are consistent with our recent transcriptomic analysis of the LG that suggests lack of E-cad expression as a characteristic feature of the IZ and that has also identified several gene products that are uniquely representative of the IP population (Girard et al., 2021).

We next characterized the cell cycle profile of these transitory cells using the Fly FUCCI system (fluorescent ubiquitination cell cycle indicator) (Zielke et al., 2011). We found that a small percentage of *CHIZ* cells were in G1, whereas the vast majority were in S and G2 (Fig. 1L; Fig. S1E), a result that was also confirmed by flow cytometric analysis (Fig. S1F). As the Fly FUCCI system is unable to distinguish between G2 and early mitotic phases, we sought to measure the occurrence of *CHIZ* cells undergoing mitosis. We found that *CHIZ* cells rarely overlap with phosphorylated Histone H3 (pH3) in the second and in the third instar (Fig. 1M,N). To estimate the ability of *CHIZ* cells to undergo mitosis, we used loss-of-function genotypes in the mitosis-promoting kinase Aurora B (AurB). Loss of this protein is expected to prevent condensation and coupling of chromosomes during mitosis leading to large nuclei with replicated chromosomes (Adams et al., 2001; Giet and Glover, 2001). Expression of *auroraB-RNAi* in the MZ resulted in *dome*+ cells with large nuclei, whereas when expressed in the IPs we did not see a change in the nuclear size of these cells (Fig. S1H-K). Furthermore, cells expressing *auroraB-RNAi* will block when they attempt to enter mitosis. As a result, the number of progenitor cells decreased dramatically when *auroraB-RNAi* was expressed in them (Fig. S1L). In contrast, *auroraB-RNAi* expressed in the *CHIZ* cells did not give rise to a change in the number of IPs (Fig. S1L). We conclude that the increase in IZ population over development is likely due to recruitment from post-mitotic progenitors that enter the IZ, which then remain largely pre-mitotic.

### IPs contribute to all mature hemocyte populations

**Fig. 2.**
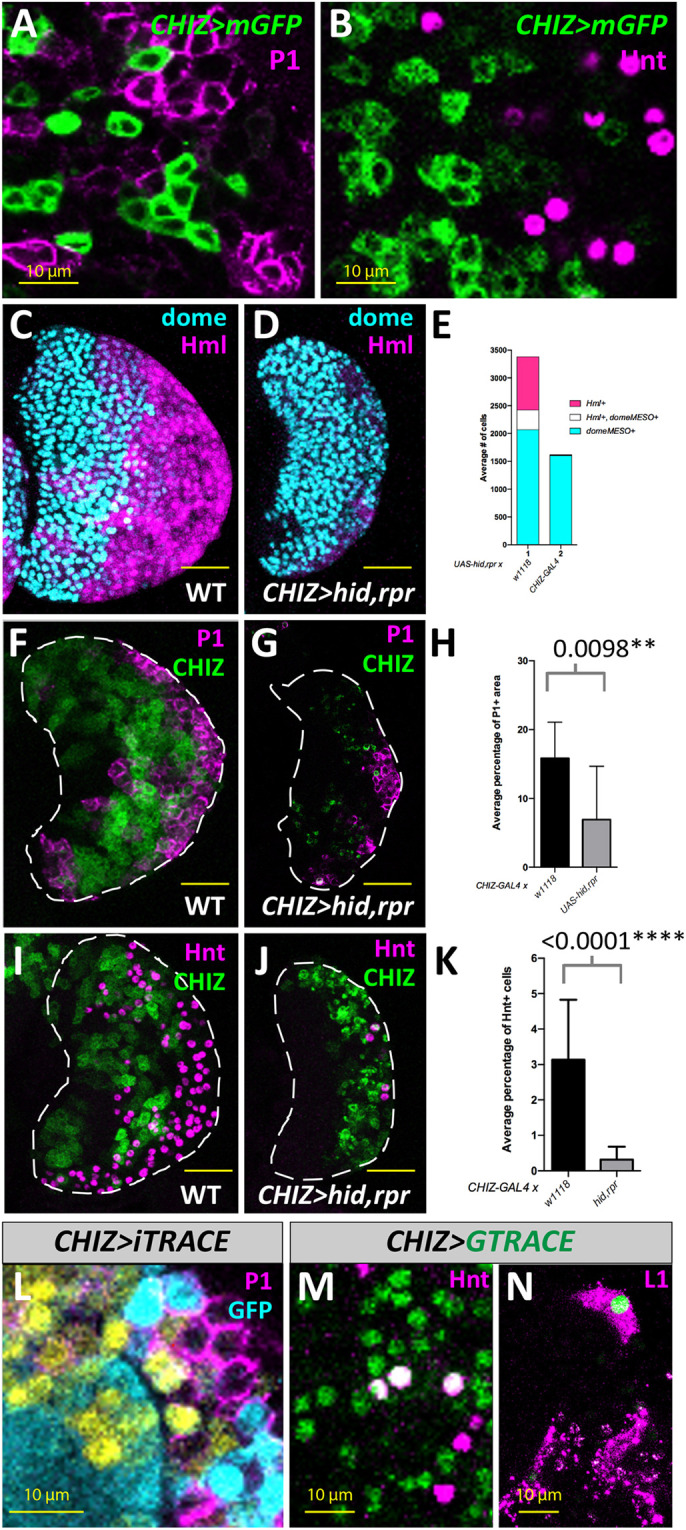
**IP cells contribute to all mature hemocyte populations.** (A) *CHIZ* cells (green) do not colocalize with mature plasmatocytes which stain for P1 (magenta). Instead, *CHIZ* cells are often seen neighboring P1-expressing cells. (B) *CHIZ* cells (green) do not stain for Hnt (magenta), a marker for crystal cells. (C) A control primary lobe without any GAL4 driver shows *dome*+ MZ cells (cyan) and *Hml*+ CZ cells (magenta) (*dome^MESO^-BFP, Hml^Δ^-DsRed, UAS-hid,rpr*). (D) Apoptosis induced in the IP population leads to a severe decrease in the *Hml*+ (magenta) population compared with *dome*+ (cyan) (*dome^MESO^-BFP, Hml^Δ^-DsRed; CHIZ-GAL4, UAS-hid,rpr*). (E) Quantitation of data shown in C and D. *n*=10 LGs for the control and *n*=8 LGs for *CHIZ>hid,rpr*. (F) Control showing non-overlap of *CHIZ* cells (green) and P1-expressing cells (magenta) (*CHIZ>mGFP*). (G) Genetic ablation of IP cells (green) leads to a reduction in P1-expressing cells (magenta). Dying *CHIZ* cells are evident as GFP puncta (green, also seen in J) (*CHIZ>mGFP, UAS-hid,rpr*). (H) Quantitation of data shown in F and G. *n*=17 LGs for the control and *n*=14 LGs for *CHIZ>hid, rpr*. (I) Control number of Hnt-expressing crystal cells (*CHIZ>mGFP*). (J) IP ablation leads to a reduction in crystal cell number (Hnt+, magenta) (*CHIZ>mGFP, UAS-hid, rpr*). (K) Quantitation of data shown in I and J. *n*=14 LGs for the control and *n*=11 LGs for *CHIZ>hid, rpr*. (L) *CHIZ* cell descendants [identified by the lack of GFP expression (cyan)] are observed to have P1 antibody staining (magenta). Live expression of *CHIZ-GAL4* is visualized in yellow (*CHIZ-GAL4; UAS-iTRACE*). (M) Crystal cells marked by Hnt antibody staining (magenta) can colocalize (white, due to overlap of green and magenta) with cells lineage traced from the *CHIZ* population (green) (*CHIZ-GAL4, UAS-GTRACE^LTO^*). (N) Cells lineage traced from the *CHIZ* population (green) can be seen expressing L1 (magenta) present in mature lamellocytes (*CHIZ-GAL4; UAS-GTRACE^LTO^*) 24 h post-injury induced at the late second instar. For LGs shown in panels L-N, lineage tracing was initiated at the mid-second instar coincident with the first appearance of *CHIZ-GAL4*+ cells and LGs were dissected at the wandering third instar. A and B are single slices from a *z*-stack, L is a maximum projection stack of 10 slices, M and N are maximum projection of three slices, and C,D,F,G,I and J are stacks of the middle third of confocal data. White dashed lines indicate the edges of LG primary lobe in F,G,I and J. Data are mean±s.d. Unpaired two-tailed Student's *t*-test. Scale bars: 25 μm (C-D,F-G,I-J); 10 μm (A-B,L-N).

Under normal conditions, *CHIZ* cells did not express P1 or Hnt (Fig. 2A,B), which are markers for mature plasmatocytes and crystal cells, respectively (Kurucz et al., 2003; Terriente-Felix et al., 2013). The IZ cells can be largely eliminated when the pro-apoptotic genes *head involution defective* (*hid*) and *reaper* (*rpr*) (Grether et al., 1995; White et al., 1994) are expressed in them. In this genetic background (*CHIZ-GAL4, UAS-hid, rpr*), the CZ population was greatly reduced (Fig. 2C-E) as were the individual numbers of P1+ plasmatocytes and Hnt+ crystal cells (Fig. 2F-K). This provides an early indication that the IPs lead to the formation of plasmatocytes and crystal cells. We confirmed this suggestion using iTRACE and G-TRACE lineage tracking constructs (Bosch et al., 2016; Evans et al., 2009) to determine the possible developmental fates of *CHIZ* cells. Analysis of the third instar LGs revealed that descendants of *CHIZ* cells are capable of committing to either plasmatocyte or to crystal cell fates (Fig. 2L,M). Lamellocytes were not observed under normal conditions, but were induced upon larval injury (Crozatier et al., 2004; Márkus et al., 2005; Rizki and Rizki, 1991, 1992). Post-injury lineage tracing experiments showed that IPs can also be fated to become lamellocytes in wandering third instar larvae (Fig. 2N). Taken together, the antibody staining, lineage tracing and ablation data showed that the IZ cells constitute a transitional population of multipotent progenitors that are capable of contributing to the CZ populations of plasmatocytes, crystal cells and lamellocytes.

### Ras/Raf activity facilitates the IP to hemocyte transition

**Fig. 3.**
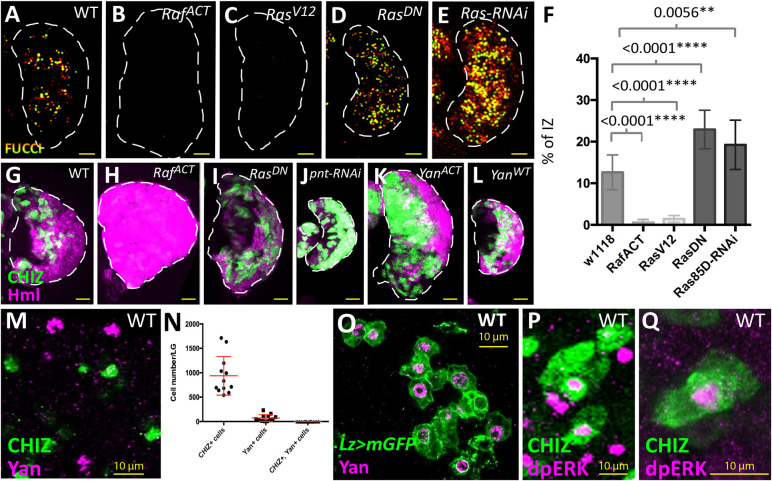
**Ras/Raf activity facilitates the IP-to-hemocyte transition.** (A-E) *CHIZ*+ IZ cells are marked with a nuclear fluorescent marker for quantification purposes (*CHIZ-GAL4, UAS-FUCCI, UAS-X*, where *X* is defined for each panel). (A) Control number of IZ cells. (B) *UAS-Raf^ACT^* leads to a loss of IZ cells. (C) *UAS-Ras^V12^* causes a similar decrease in IZ cells as B. (D) An increase in IZ cells is apparent when *CHIZ-GAL4* drives *UAS-Ras^DN^.* (E) Increased IZ cells are present when expressing *UAS-Ras85D-RNAi* in IZ cells. (F) Fraction of *CHIZ*+ cells in LGs represented in A-E. Data are mean±s.d. *n*=11, *n*=14, *n*=14, *n*=12 and *n*=12 LGs for each dataset in sequence, respectively. Unpaired two-tailed Student's *t*-test. (G-L) *CHIZ*+ IZ cells are marked in green and *Hml*+ CZ cells are labeled in magenta (*CHIZ-GAL4, UAS-mGFP; Hml^Δ^-DsRed; UAS-X*, where *X* is defined for each panel). (G) Wild type. (H) *UAS-Raf^ACT^* causes an extreme expansion of the CZ and loss of IZ. (I) *UAS-Ras^DN^* increases the proportion of IZ cells and leads to a decrease in CZ cells. (J) *UAS-pnt-RNAi* causes a large increase in proportion of IZ cells and very few CZ cells. (K) *UAS-Yan^ACT^* causes an increased IZ and reduced CZ. (L) *UAS-Yan^WT^* does not result in a shift in the general proportion of IZ to CZ as seen in K. Images of LGs in A-E and G-L are maximum projections of the middle third of a Z-stack. (M) IZ cells (green) do not directly colocalize with nuclear Yan protein (magenta) (*CHIZ>dsGFP*). (N) Data from *CHIZ>dsGFP* LGs showing lack of any significant overlap between *CHIZ*+ and Yan+ cells. *n*=12 LGs. (O) Crystal cells (green) express nuclear Yan protein (magenta) (*Lz-GAL4, UAS-mGFP*). (P,Q) A subset of IZ cells (green) shows nuclear dpERK staining (magenta) (*CHIZ>mGFP*). Panels M,O,P and Q are maximum projection stacks of three slices of a *z*-stack. Data are mean±s.d. Unpaired two-tailed Student's *t*-test. Scale bars: 25 μm (A-E,G-L); 10 μm (M,O-Q).

We next investigated the function of known molecular pathways in the transition between IPs and maturing hemocytes. Two major signaling pathways, Ras/Raf and Notch, operate during LG development (Crozatier et al., 2004; Dragojlovic-Munther and Martinez-Agosto, 2013; Krzemien et al., 2007; Mondal et al., 2011, 2014; Mukherjee et al., 2011) but any specific role they might play in the IZ population has not been explored. Later we discuss the role of the Notch pathway in the IPs. Activation of the Ras/Raf/MAPK pathway in *Drosophila* leads to the phosphorylation of Pointed (Pnt) and Yan (Aop), both ETS family proteins that function as downstream transcriptional activator and repressor, respectively (Brunner et al., 1994; Lai and Rubin, 1992; Nusslein-Volhard et al., 1984; O'Neill et al., 1994). Activated forms of Ras or Raf expressed specifically in the IPs caused a reduction in the number of the IPs (Fig. 3A-C,F) and, reciprocally, inhibition of the Ras pathway increased the IZ population (Fig. 3A,D-F). Manipulation of Ras/Raf led to a shift in cell cycle of the IPs. Their inhibition led to an increase in the number of IPs in the G2 phase of the cell cycle and their overactivation led to a higher percentage of IPs in S phase (Fig. S2A). Concomitantly, we observed an increase in the number of *Hml*+ cells upon activation of the Ras/Raf pathway and a decrease in this population upon loss-of-function of this pathway (Fig. 3G-L). The loss-of-function phenotype was strikingly apparent when *pnt* expression was knocked down in the *CHIZ* cells (Fig. 3J) or when a constitutively active version of Yan (Yan^ACT^) (Rebay and Rubin, 1995), expected to block Ras pathway signals, was expressed in the IPs (Fig. 3K). This latter result was not phenocopied if the overexpressed version of Yan was wild type (Yan^WT^, not constitutively activated) (Fig. 3L). The phenotypic distinction between Yan^ACT^ and Yan^WT^ overexpression further supports the presence of an activated Ras pathway that will cause degradation of the wild-type but not the activated version of Yan. Immunohistochemical localization showed no detectable Yan protein in the IPs (Fig. 3M,N; of 938 average *CHIZ*+ cells, 0.17% appear to colocalize with Yan, Fig. S2B). A previous report suggesting that Yan+ cells are part of the IZ preceded the discovery of an IP-specific marker (Tokusumi et al., 2011). Instead, the Yan protein was detected in crystal cells and *yan-RNAi* expressed in crystal cells eliminated all Yan expression in the LG (Fig. 3O; Fig. S2C,E-G′). Finally, a subset of *CHIZ* cells expressed the nuclear form of dp-ERK (active MAPK), but we note that dp-ERK was additionally observed sporadically throughout the LG (Fig. 3P,Q; Fig. S2D). Altogether, these results suggest that cells of the IZ require Ras/Raf signaling to exit the *CHIZ* state and that in the absence of such a signal, the IP cells are held back in their transitional *CHIZ* state.

### IP cells induce crystal cell formation mediated by the Notch pathway

**Fig. 4.**
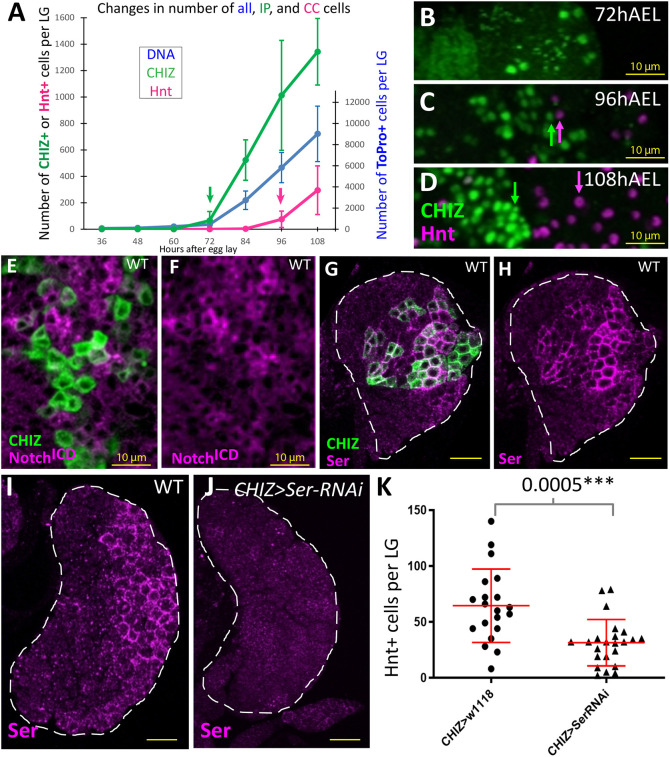
**IP cells induce crystal cell formation mediated by the Notch pathway.** (A-D) Genotype is *CHIZ-GAL4, UAS-dsGFP*. *CHIZ* cells are green (GFP) and crystal cells are magenta (Hnt). (A) Numbers represent measurement of two combined primary lobes from a single LG. Quantification of the raw number of *CHIZ*+ (green), crystal cells (magenta) and total number of cells (DNA marked by ToPro, blue) in LGs of developmentally synchronized larvae. The first significant appearance of *CHIZ* cells is at 72 hAEL (green arrow), and the first significant appearance of crystal cells is later, at 96 hAEL (magenta arrow). *n*=6, *n*=6, *n*=5, *n*=8, *n*=10, *n*=14 and *n*=5 LGs for each sample point in sequence, respectively. (B-J) All images are single slices of confocal data. (B) Section from a 72 hAEL primary lobe showing the first appearance of *CHIZ* cells. (C) At 96 hAEL the earliest crystal cells (magenta arrows) are usually seen neighboring *CHIZ* cells (green arrows). (D) At 108 hAEL wandering third instar primary lobes have numerous crystal cells (magenta arrow) distant from *CHIZ* cells (green arrow). (E-H) Genotype is *CHIZ-GAL4, UAS-mGFP*. (E,F) *CHIZ* cells (green) do not colocalize with high levels of N^ICD^ protein (magenta) observed in neighboring cells. (G,H) *CHIZ* cells (green) colocalize with high levels of Serrate protein (Ser; magenta) in early third instar. (I) Control showing Ser protein expression (magenta) in early third instar LG. (J) Ser protein (magenta) is absent in early third instar when *Ser-RNAi* is driven by *CHIZ-GAL4* (*CHIZ-GAL4, UAS-Ser-RNAi*). (K) The number of crystal cells (Hnt+) per LG decreases when *Ser-RNAi* is expressed in IP cells (*CHIZ-GAL4, UAS-Ser-RNAi*, *n*=23 LGs) compared with control (*CHIZ-GAL4*, *n*=20 LGs). White dashed lines indicate the edges of LG primary lobe in G-J. Data are mean±s.d. Unpaired two-tailed Student's *t*-test. Scale bars: 10 μm (B-F); 25 μm (G-J).

*CHIZ* cells appeared at 72 h after egg lay (hAEL; late second instar) before the appearance of the first crystal cells between 84 and 96 hAEL (Fig. 4A; early to mid-third instar). At 72 hAEL, *CHIZ*+ but not Hnt+ cells were seen (Fig. 4B). At 96 hAEL, crystal cells were primarily detected in the immediate vicinity of the *CHIZ* cells (Fig. 4C). These two cell types separated from each other by 108 hAEL (Fig. 4D; wandering third instar). These data suggest a close temporal and spatial relationship between these two cell types during early stages of hemocyte differentiation.

Past studies have shown that Serrate/Notch (Ser/N) signaling is important for crystal cell specification in the LG (Mukherjee et al., 2011; Terriente-Felix et al., 2013), and therefore we investigated the Notch pathway in the context of IP function. An antibody raised against the intracellular domain of Notch (Notch^ICD^) detected low level expression of Notch throughout the LG, with the highest level of staining seen in cells positioned adjacent to *CHIZ* cells (Fig. 4E,F). Ser expression is highly dynamic. Virtually all of the earliest appearing *CHIZ* cells expressed high levels of Ser in the mid-second instar. Although this colocalization of IPs and Ser+ cells continued through mid-third instar, a number of non-overlapping cells were evident (Fig. 4G,H). Ser expression was greatly attenuated and virtually undetectable in the wandering third instar, with no correspondence between a few residual low Ser-expressing cells and the large number of *CHIZ* cells (Fig. S3A,A′). Importantly, knock-down of *Ser* specifically in *CHIZ* cells eliminated Ser protein expression in all cells of the LG (Fig. 4I,J). This indicates that all Ser-expressing cells in the LG transition through a *CHIZ*-state at some point in their development and that the dynamic pattern of Ser is a reflection of the tight temporal control of its expression. Furthermore, *Ser-RNAi* expressed in *CHIZ* cells caused a significant reduction in the number of crystal cells without decreasing the number of IPs themselves (Fig. 4K; Fig. S3B). We conclude that the IP-specific Ser expression promotes induction of crystal cell fate in the neighboring *Hml*+ cells. However, we postulate that the dynamic nature of Ser expression limits the number of neighbors that can take on a crystal cell fate.

## DISCUSSION

Progenitor and differentiated cell types have been well described in *Drosophila* hematopoiesis (reviewed by Banerjee et al., 2019). Genetic evidence suggested that certain cells have an intermediate characteristic in that they express some progenitor as well as mature cell markers (Sinenko et al., 2009; Krzemien et al., 2010). Although these cells could be identified during *Drosophila* hematopoiesis owing to their overlapping expression patterns, the absence of tools to directly detect such populations has thus far prevented a detailed analysis of these transitional cells. These cells have been designated IPs and they bridge MZ progenitors with the CZ hemocytes. In this article, we use a Split GAL4 strategy to generate *CHIZ-GAL4* that allows us to identify and genetically manipulate IPs. The IPs of the IZ represent a unique cell type that have some characteristics that are distinct from and others that are similar to the cells of the MZ and CZ. For example, IPs express *dome*, but not E-cad, both of which are MZ markers. Similarly, IPs express *Hml*, but not the maturity markers P1 (plasmatocytes) and Hnt (crystal cells). Interestingly, the IP cells share the property of multipotency with cells of the MZ in that both can contribute to all three populations of mature hemocytes. Importantly, we believe IPs to be a unique cell type, as their numbers can be expanded or reduced upon genetic manipulation as shown, for example, with modulation of the Ras pathway. In addition, bulk and single cell RNA-seq data obtained recently in the laboratory identifies several genes that are highly enriched within IPs when compared with their expression in all other cell types in the LG (Girard et al., 2021). In future studies, these will serve well as specific IZ markers and provide further functional relevance for this population.

The MZ cells are fairly quiescent (Jung et al., 2005; Krzemien et al., 2010; Lebestky et al., 2003); they are largely held in G2 (Sharma et al., 2019), and will undergo mitosis in a limited number of cells. In contrast, IP cells are found in G1, S and G2 but with a very limited extent of mitosis. We propose that before entering the IP state, a *dome*+ progenitor is released from G2 and it undergoes mitosis. Subsequently, *Hml* is initiated and continues to be expressed as IPs progress through G1, S and G2. At this point *dome* expression ceases, thus ending the *CHIZ*-state. The *dome*-negative post-*CHIZ* cell likely undergoes a round of mitosis before it progresses to a differentiated state. The IPs are multipotent and contribute to all of the three mature hemocyte populations. We should note that the data presented in this study do not preclude the possibility that a few of the hemocytes might form by a parallel mechanism that does not involve the IPs.

As in many developmental systems, entry into a proliferative state and fate determination are intimately intertwined and this applies as well to the transition from the IZ to the CZ. We presume that a mitotic event must closely follow exit from the IP state and is linked to differentiation into a hemocyte. We also know that the Ras/Raf pathway is required for exit out of the IP state. In other systems, Ras/Raf activity has largely been associated with proliferation (reviewed by Bryant et al., 2014; Karnoub and Weinberg, 2008; Lu et al., 2016), but in *Drosophila*, this pathway often governs cell fate determination, as seen, for example, during the development of the eye imaginal disc (Flores et al., 2000; Freeman, 1996; Nagaraj and Banerjee, 2007; Simon et al., 1991). Thus, it remains uncertain at the present moment whether Ras/Raf initiates the mitotic process and this allows differentiation signals to be sensed to turn on markers, or whether another mechanism controls the entry into mitosis and Ras is responsible for turning off a marker such as *dome*. In a manner similar to that seen in other well-defined developmental situations in *Drosophila*, the Ras/Raf and Notch pathways play dueling roles in the post-*CHIZ* stage of defining cell fate. The IPs express Ser in a dynamic pattern and induce neighbors to take on a crystal cell fate. The expression of Ser is downregulated after the mid-third instar, and its restricted spatial and temporal pattern of expression limits crystal cell number. Crystal cells do not have active Ras signaling as established by their expression of the Yan protein. The Ras/Raf signal promotes plasmatocyte fate, whereas crystal cells are dependent on Notch signaling. Upstream events that activate Ras in the IPs are currently unknown and will be of great interest for future investigation. It is possible that a canonical ligand-dependent RTK may be involved; however, other autonomous molecular mechanisms such as changes in metabolism could feed into Ras (Girard et al., 2021).

IPs may provide an opportunity to synchronize the assignment of cell fate during normal development, and maintain plasmatocytes and crystal cells in a stereotypical ratio (Ghosh et al., 2015; Lebestky et al., 2000; Leitao and Sucena, 2015; Tepass et al., 1994). It is also likely that IPs have unique signaling functions as inferred from their regulation of Ser expression to induce direct neighbors to take on a crystal-cell fate. It is interesting to note that this transitional population acts autonomously as multipotent progenitors while they also non-autonomously induce one of the specific blood cell fates. Investigation into the expression of receptors and ligands in IPs will expand our current understanding of the role these cells play in regulating the balance between progenitors and the various determined blood cell types during homeostasis. If all progenitors in the MZ were to directly differentiate into mature hemocytes without going through the buffer zone provided by the IPs, then a relatively steady pool of progenitors will be difficult to preserve, and the spatio-temporal order of hemocyte specification will not be maintained. Under stress conditions or immune challenge this buffer could be altered in favor of faster production of hemocytes at the cost of progenitor number.

The experimental strategy used to develop *CHIZ-GAL4* has been successfully adapted for identifying cell types based on the co-expression of other genes in *Drosophila*, particularly in the nervous system (Jenett et al., 2012; Luan et al., 2006; Pfeiffer et al., 2008, 2010). There is nothing about this strategy that is *Drosophila*-specific and one hopes that its most useful application might be to uncover cryptic cell types in the context of the significantly more complex transitions described in mammalian hematopoietic development.

## MATERIALS AND METHODS

### *Drosophila* stocks and husbandry

The following *Drosophila* stocks were used for this study: w*^1118^* (U.B.), *Hml^Δ^-DsRed.nls* (Katja Brüeckner, University of California, San Francisco, USA), *dome^MESO^-GFP.nls, Hml^Δ^-DsRed.nls/CyO* (U.B.), *dome^MESO^-GAL4-AD, Hml^Δ^-GAL4-DBD* (*CHIZ-GAL4*, developed in Banerjee Lab for this paper, see below), *UAS-mGFP* (II) (U.B.), *CHIZ-GAL4, UAS-mGFP* (U.B.), *dome^MESO^-BFP, Hml^Δ^-DsRed, Hh-GFP/FM7* (U.B.), *UAS-dsGFP* (II) (Brian McCabe, Brain Mind Institute at EPFL in Lausanne Switzerland), *UAS-FUCCI* [BL55722, Bloomington Drosophila Stock Center (BDSC)], *UAS-2xEGFP* (U.B.), *dome^MESO^-GAL4* (U.B.), *AuroraB-RNAi* (BL28691, BDSC), *UAS-iTRACE* (BL66387, BDSC), *UAS-GTRACE^LTO^* (U.B.), *dome^MESO^-BFP, Hml^Δ^-DsRed, Hh-GFP, UAS-hid, rpr/FM7* (U.B.), *UAS-hid, rpr* (U.B.), *CHIZ-GAL4, UAS-mGFP; Hml^Δ^-DsRed* (U.B.), *UAS-Raf^ACT^* (BL2033, BDSC), *UAS-Ras^V12^* (Gerald M. Rubin, Janelia Research Campus, Howard Hughes Medical Institute, Ashburn, USA), *UAS-Ras^DN^* (U.B.), *UAS-Ras85d-RNAi* (BL29319, BDSC), *UAS-pnt-RNAi* (BL31936, BDSC), *UAS-Yan^ACT^* (BL5789, BDSC), *UAS-Yan^WT^* (BL5790, BDSC), *Lz-GAL4, UAS-mGFP* (BL6314, BDSC), *UAS-Yan-RNAi* (BL34909, BDSC), *UAS-Yan-RNAi* (BL35404, BDSC) and *Ser-RNAi* (Vienna Drosophila Resource Center, 27172). All stocks were maintained at room temperature or 18°C. All genetic crosses, with the exclusion of the staged larval time course experiment described below, were raised at 29°C for maximum GAL4-UAS efficiency. All flies were raised on standard *Drosophila* fly food with a recipe containing dextrose, corn meal and yeast.

### Development of *CHIZ-GAL4* driver

The *dome^MESO^* enhancer (*dM-*forward primer: 5′-CACCCGTCTACCGCGATTCCAAGCACATCCG-3′; *dM*-reverse primer: 5′-GGATCCAAAATACCCGATGTAAAATCG-3′) and *Hml^Δ^* enhancer (*Hml^Δ^*-forward primer: 5′-CACCGGTACCCAAAAGTTATTTCTG-3′; *Hml^Δ^*-reverse primer: 5′-GTTTAATTGTATACACAGGAAAATC-3′) were amplified from *Drosophila* genomic DNA and ligated into the pENTR™/D-TOPO™ vector (Invitrogen, K240020) for Gateway cloning. Each entry vector was ligated into the pBPp65ADZpUw (Addgene plasmid #26234) and pBPZpGAL4DBDUw (Addgene plasmid #26233) destination vectors using the LR ligase (Invitrogen, 11791020) to generate the desired vectors (*dome^MESO^-p65-AD* and *Hml^Δ^-pGAL4-DBD*). These vectors were sent to BestGene Inc for microinjection. Transgenic flies were generated by PhiC31 integrase-mediated site-specific transgenesis. The *Hml^Δ^-pGAL4-DBD* was integrated into the 51C locus of the *Drosophila* genome (Injection Stock, 24482NF), whereas the *dome^MESO^-p65-AD* was integrated into the 58A locus (Injection Stock, 24484NF). The transgenic *Drosophila* lines were crossed to generate *dome^MESO^-p65-AD, Hml^Δ^-pGAL4-DBD* stable lines through homologous recombination.

### Lymph gland dissection and immunohistochemistry

Larval head complexes were dissected on a silicon dissecting dish in chilled 1× PBS. Head complexes including mouth hooks, eye-antennal discs, brain and ventral nerve cord, and LGs were immersed in fixation solution (4% formaldehyde in 1× PBS) for 25 min. After fixation, samples were washed three times for 10 min in LG wash buffer (0.4% Triton in 1× PBS). Samples were incubated in 10% normal goat serum in 1× PBS blocking solution for 10-30 min then incubated in primary antibody overnight at 4°C. Samples were washed in LG wash buffer, then incubated in secondary antibody for 2-4 h at room temperature. After washing off the secondary antibody in LG wash buffer, ToPro-3 dye (Invitrogen) was incorporated at a 1:1000 concentration for 7-10 min to visualize nuclei of tissues. After a final wash step, samples were immersed in Vectashield anti-fade mounting media, placed on a glass slide, and LGs were isolated from the head complexes and mounted. Samples were covered with a glass coverslip which was sealed with clear nail polish. Slides were stored at 4°C until imaged.

Primary antibodies used in this study include: rabbit anti-GFP (1:100, Thermo Fisher Scientific, A-11122), rat anti-E-cad [1:20, Developmental Studies Hybridoma Bank (DSHB), DCAD2], rabbit anti-PH3 (1:400, Cell Signaling Technology, 9706S), mouse anti-P1 [1:100, Istvan Ando, Institute of Genetics, Biological Research Center (BRC), Szeged, Hungary], mouse anti-Hnt (1:200, DSHB, 1G9), mouse anti-Notch^ICD^ (1:100, DSHB, C19.9C6), rat anti-Serrate (1:1000, Ken Irvine, Waksman Institute and Department of Molecular Biology and Biochemistry, Rutgers University, Piscataway, NJ, USA), mouse anti-Yan (1:100, DSHB, 8B12H9), rabbit anti-dpERK (1:100, Cell Signaling Technology, 4370) and anti-L1 (1:10, Istvan Ando). Secondary antibodies used in this study were purchased from Invitrogen and include: donkey anti-mouse AlexaFluor405 (A48257), donkey anti-mouse AlexaFluor488 (R37114), donkey anti-mouse AlexaFluor555 (A31570), goat anti-mouse Alexa-Fluor633 (A21050), donkey anti-rabbit AlexaFluor488 (A21206), donkey anti-rabbit AlexaFluor555 (A31572), goat anti-rat AlexaFluor555 (A21434), donkey anti-rat Cy3 (AB_2340666) and donkey anti-mouse Cy3 (AB_2340813) from Jackson Scientific. Secondary antibodies were used at a 1:100-1:2000 dilution dependent on the strength of the primary antibody.

### Staged larval lymph gland dissections

For data collected presented in Fig. 4A-D, larvae were synchronized within 1 h of each other in 12 h phases. Then 100-200 mated flies (*CHIZ-GAL4*×*UAS-dsGFP*) were maintained in collection chambers at 25°C and allowed to lay embryos on plates containing ethyl acetate (EA) media. After a 12 h collection period, new EA plates were provided to the adults in collection chambers. The embryos on the old EA plates were incubated at 25°C for 24 h. After this incubation time, hatched larvae were cleared from the plate using a paintbrush and the remaining unhatched embryos were incubated for 1 h at 25°C. After 1 h, newly hatched larvae were transferred with a paintbrush to a fresh vial of food. Five larvae were placed in each vial. Vials were incubated at 25°C until samples from all time points were dissected and processed for immunohistochemistry on the same day.

### Microscopy and image processing

All samples were imaged using a Zeiss LSM-880 confocal microscope using a *z*-stack technique with 1.88 μm slice thickness. Images were processed using ImageJ. Unless otherwise noted in the figure legends, images of LGs are a maximum intensity projection of the stack of the middle third of the samples. Using this technique allows for visibility of the inside of the LG which can become obfuscated by the expression of antigens localized to the CZ region in a maximum intensity projection of the entire LG.

### Data quantification

All quantifications were performed using Imaris data analysis software by Bitplane to quantify *z*-stacks of entire LGs. Briefly, LGs were contoured and fluorescent channels were masked to restrict quantifications to both primary lobes. To label and count nuclei, a spots filter was applied based on ToPro DNA dye incorporation. The DNA+ spots were then filtered against additional fluorescent channels to quantify specific cell types including *Hh-GFP*, *domeMESO-BFP*, *CHIZ>mGFP*, *Hml-DsRed*, *CHIZ>dsGFP*+, PH3+ or Hnt+. FUCCI+ cells, *CHIZ*+ PH3+ cells and *CHIZ*+ Yan+ cells were identified by positively filtering for additional fluorophores. The percentage of the LG occupied by these particular cell types was determined by dividing the number of cells of interest by the total number of nuclei per LG, then multiplying by 100. When quantifying the volume of the LG and volume of P1+ fluorescence for data presented in Fig. 2H, the surfaces filter was first applied based on ToPro DNA dye incorporation and then extended to fill in the volume of both primary lobes. A second volume measurement was made using the surfaces filter for P1+ fluorescence. The percent of the LG occupied by P1+ fluorescence was calculated by dividing the volume of P1+ fluorescence by the total LG volume, then multiplying by 100. All *P*-values presented represent unpaired two-tailed Student's *t*-tests to determine statistical significance.

### Flow cytometry

*CHIZ-GAL4, UAS-FUCCI* LGs were dissected in 1× modified dissecting saline solution (MDSS; 9.9 mM HEPES-KOH, 137 mM NaCl, 5.4 mM KCL, 0.17 mM NaH_2_PO_4_, 0.22 mM KH_2_PO_4_, 3.3 mM glucose, 43.8 mM sucrose, pH 7.4) and immediately submerged in Schneider's S2 media in a glass watch glass on ice. Isolated LGs were washed once with 1× MDSS, and then the 1× MDSS was removed. We then added 200 μl of heat activated Papain solution (100 units/ml) to LGs, which were then moved to an Eppendorf tube. Samples were covered in foil and incubated in Papain solution while shaking at 25°C for 15 min. During incubation, tubes were removed twice to pipette up and down to break up tissue. Papain solution was inactivated by the addition of 500 μl cold S2 media. Tissue was centrifuged at 3000 rpm (840 ***g***) for 5 min. Supernatant was removed and 1 ml of 1% formaldehyde was added to the cell pellet. Cells were shaken in fixative at 4°C for 30 min. Cells were spun down at 3000 rpm (840 ***g***) for 5 min and supernatant was removed. Cell nuclei were labeled by incubating pellet at room temperature for 30 min in NucBlue live cell stain Ready Probes Reagent (Invitrogen, Hoechst 33342 Special Formulation). The sample was transferred to a round-bottom polystyrene tube and samples were run through a BD LSRII FACS analyzer. Gates for cell fluorescence were standardized using single fluorophore controls. This experiment was replicated five times using 50-85 LGs per round.

## Supplementary Material

Supplementary information

Reviewer comments
