# Supplementary material for: Intermediate progenitor cells provide a transition between hematopoietic progenitors and their differentiated descendants
Source: Development. 2021 Dec 17;148(24):dev200216. doi: 10.1242/dev.200216 (PMC8722385; doi:10.1242/dev.200216)
Supplement: Supplementary information [file develop-148-200216-s1.pdf]

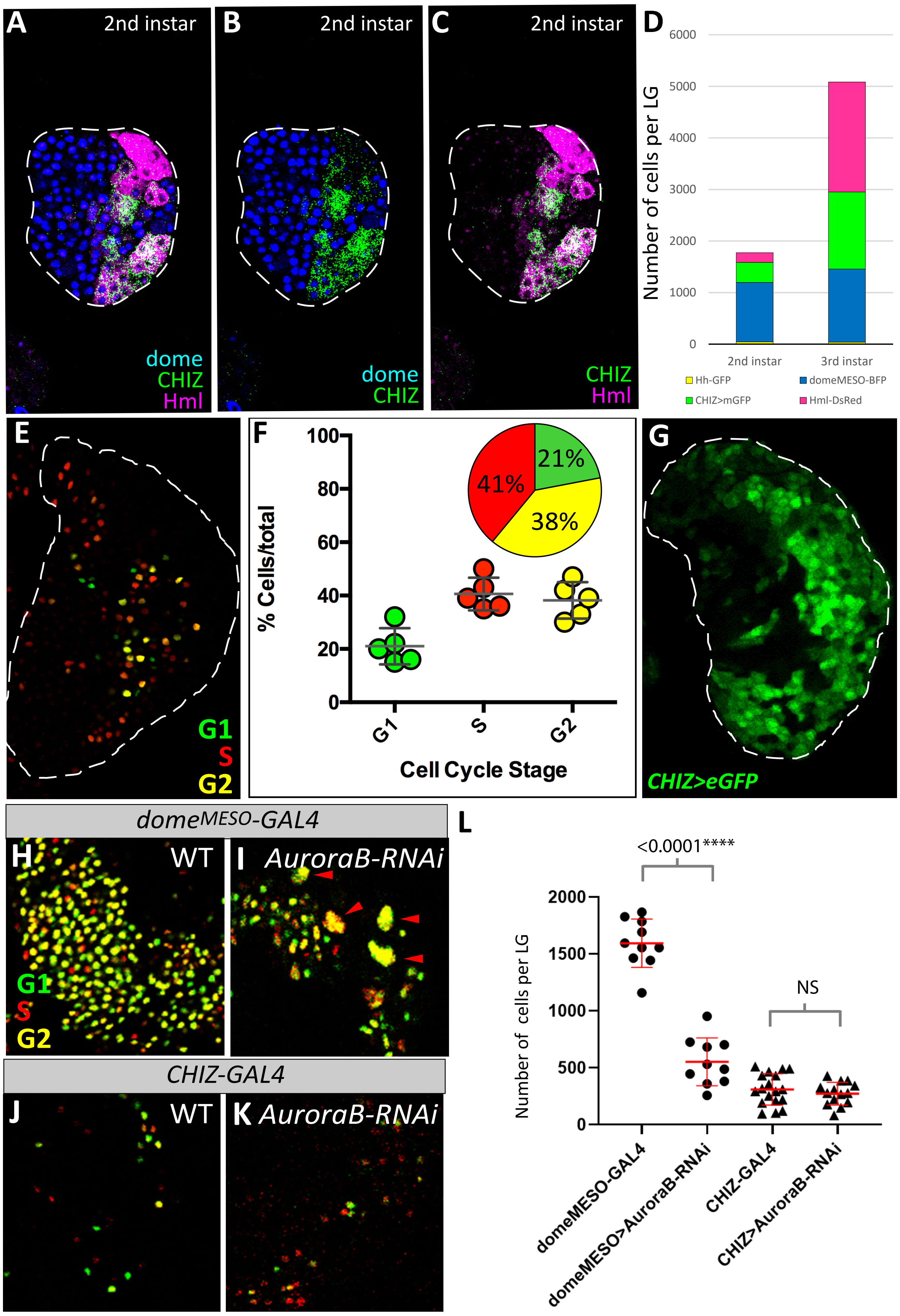

Development • Supplementary information

Fig. S1. Further characterization of the intermediate zone cell population. (A-C) A second instar lymph gland with fluorescently labeled zones (*dome*^{MESO}*-BFP, Hml*$^{\Delta}$*-DsRed; CHIZ-GAL4, UAS-mGFP*) shows *CHIZ-GAL4* expression (green) juxtaposed between the MZ (*dome*+, blue) and CZ (*Hml*+, magenta). **A-C** represent the same lymph gland. For clarity, the *Hml* (magenta) channel is omitted in **B**, and the *dome* (blue) channel is omitted in **C**. **(D)** Quantification of *Hh-GFP*, *dome*^{MESO}*-BFP*, *CHIZ>mGFP*, and *Hml-DsRed* cells in the primary lobes of second and third instar lymph glands. At second instar, the respective percentages of cells in the PSC, MZ, IZ, and CZ are 2, 65, 22, and 11 whereas in the third instar the percentages are 1, 28, 30, and 42. **(E)** IPs can be in G1 (green), S (red), or G2 (yellow) phases of the cell cycle (*CHIZ-GAL4; UAS-FUCCI*). **(F)** Flow cytometric analysis of IPs indicates the majority of IPs are distributed equally between S (red) and G2 (yellow) with a smaller percent of cells in G1 (green) (*CHIZ-GAL4; UAS-FUCCI*). **(G)** Extended perdurance of strong and long-lived fluorophores such as eGFP (green) do not properly represent the specificity of *CHIZ-GAL4* expression. Such fluorophores perdure into the CZ region and fail to follow the transitory nature of the IPs (*CHIZ-GAL4; UAS-2xeGFP*). **(H-K)** Nuclear size-based assay for M-phase cells **(H)** Nuclei of progenitors marked by a cell cycle indicator (*dome*^{MESO}*-GAL4, UAS-FUCCI*). **(I)** *dome*+ nuclei attempting to enter M-phase at the edge of the MZ become enlarged in size when mitosis is blocked by loss of *AuroraB* (red arrowheads) (*dome*^{MESO}*-GAL4; UAS-FUCCI, UAS-AuroraB-RNAi*). **(J)** Nuclei of IP cells marked by a cell cycle indicator (*CHIZ-GAL4; UAS-FUCCI*). **(K)** Due to lack of M-phase cells, nuclear size of IPs is not affected upon loss of *AuroraB* (*CHIZ-GAL4; UAS-FUCCI, UAS-AuroraB-RNAi*). **(L)** Quantification of the number of progenitors and IP cells. *UAS-FUCCI* is used here as a marker of all cells where the driver is active. Knockdown of *Aurora B* in *domeMESO*+ cells causes a significant reduction in the number of progenitors (*dome*^{MESO}*-GAL4; UAS-FUCCI, UAS-AuroraB-RNAi, n=10* LGs) whereas knockdown of Aurora B in the IPs (*CHIZ-GAL4; UAS-FUCCI, UAS-AuroraB-RNAi, n=14* LGs) has no effect on the IP population numbers. The two comparisons are made with their respective controls that lack *AuroraB-RNAi*. *n*=10 LGs for *dome-GAL4, UAS-FUCCI* and *n*=18 LGs for *CHIZ-GAL4, UAS-FUCCI*. The standard deviation and mean values are indicated. White dashed lines indicate the edges of lymph gland primary lobe in **A-C, E** and **G**. Yellow scale bars represent 25 µm in **A-C**, **E**, and **G** and 10 µm in **H-K**.

Development • Supplementary information

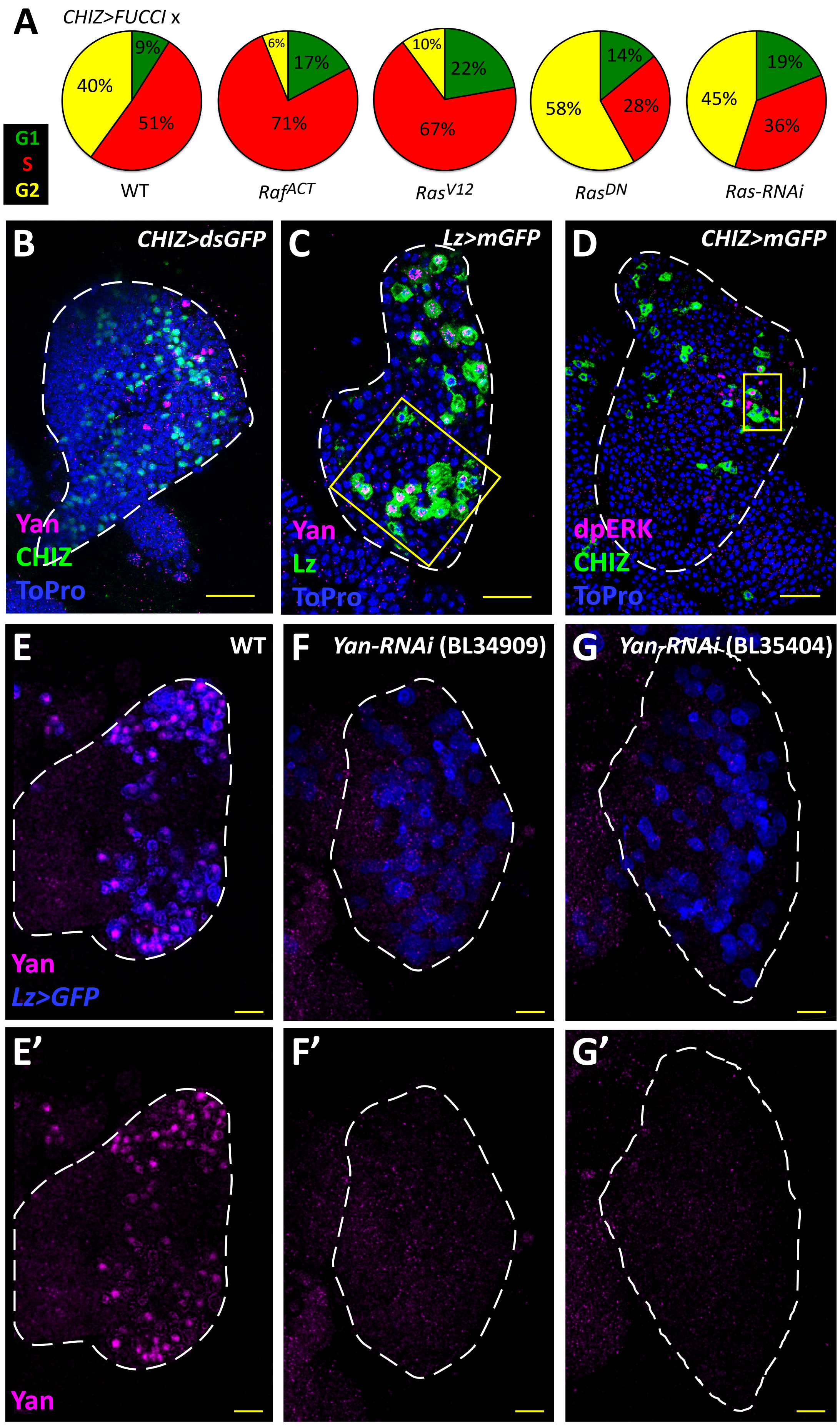

Fig. S2. Ras/Raf regulation of cell cycle in IPs and crystal cell-specific expression of Yan. (A) Pie charts representing the average percent of *CHIZ-GAL4* expressing cells in primary lobes of the lymph glands displayed in **Figure 3A-E** that are in G1 (green), S phase (red), and G2/early M phase (yellow) as assessed by the expression of the *Fly FUCCI* indicator. *n*=11, *n*=14, *n*=14, *n*=12, and *n*=12 LGs for each data point in sequence respectively. **(B)** Full primary lobe from which high magnification image in **Figure 3M** was taken. IZ cells (green) do not directly co-localize with nuclear Yan protein (magenta) (*CHIZ>dsGFP*). **(C)** Full primary lobe from which high magnification image (area indicated by the yellow box) in **Figure 3O** was taken. Crystal cells (green) express nuclear Yan protein (magenta) (*Lz-GAL4*, *UAS-mGFP*). **(D)** Full primary lobe from which high magnification image (area indicated by the yellow box) in **Figure 3P** was taken. A subset of IZ cells (green) show nuclear dpERK staining (magenta) (CHIZ>mGFP). **(E, E')** Control showing Yan staining (magenta) colocalized with crystal cells (blue) throughout the primary lobe.

Development • Supplementary information

(F-G') Expression of two separate *Yan-RNAi* constructs, BL34909 **(F,F')** and BL35404 **(G,G')** driven by *Lz-GAL4*. Lz+ crystal cells form **(F, G)**, even though they are devoid of nuclear Yan accumulation **(F', G')**. White dashed lines in **B-G'** indicate the outer edges of the primary lobe. Yellow scale bars in **B-G'** represent 25 μm.

Development • Supplementary information

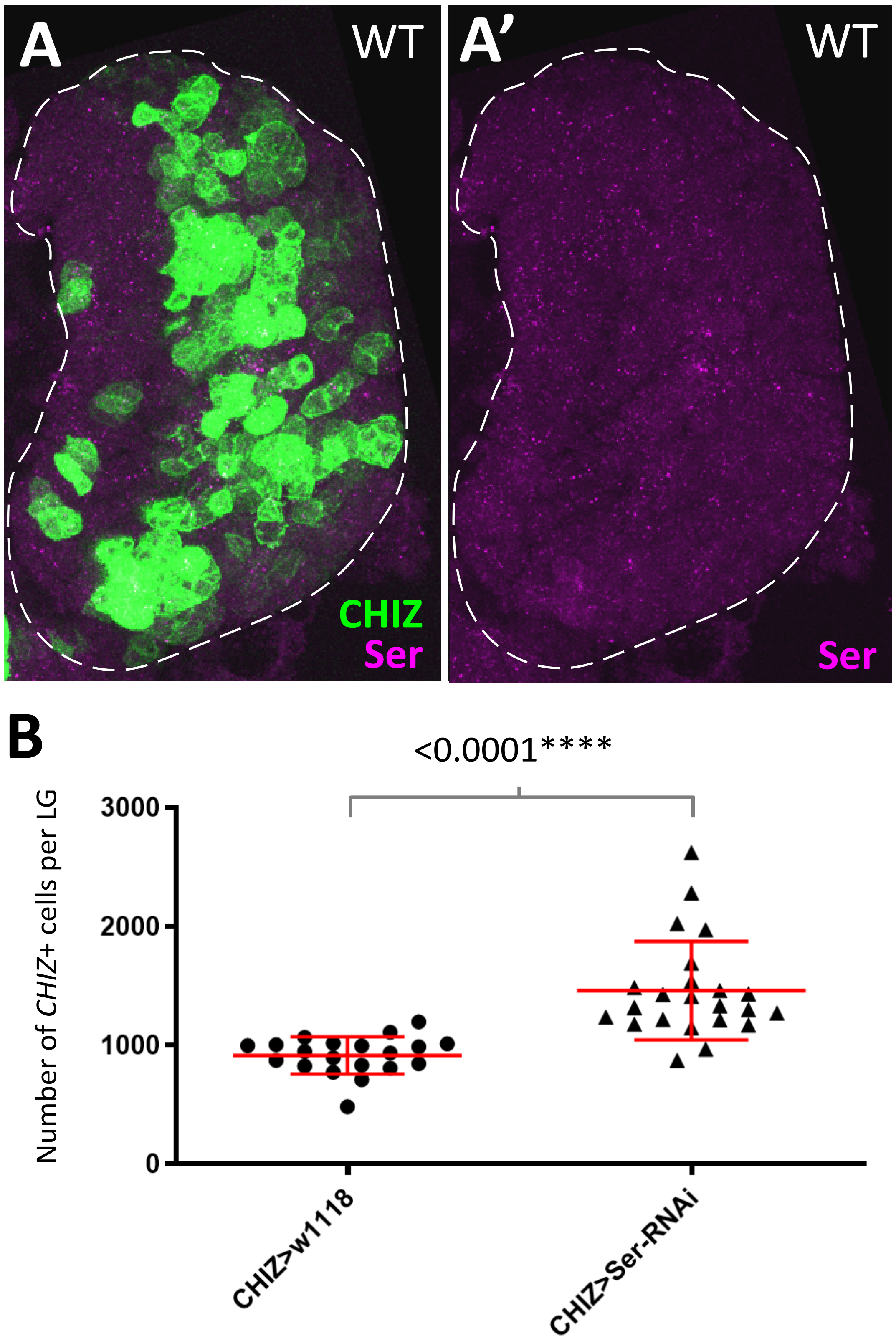

Fig. S3. Relationship between IPs and Serrate expression. (A, A’) Control wandering late third instar lymph gland shows Serrate staining is virtually absent (magenta) and no longer has a correlation with *CHIZ* cells (green). Images are a maximum projection of the middle third of a confocal Z-stack. (*CHIZ-GAL4, UAS-mGFP*). Yellow scale bars represent 25 µm. **(B)** Quantification of *CHIZ*+ cells upon Ser knockdown (*CHIZ-GAL4, UAS-Ser-RNAi, n=23* LGs) compared to control (*CHIZ-GAL4, n=20* LGs). Standard deviation and mean are indicated.

Development • Supplementary information
