# Supplementary material for: Intermediate progenitor cells provide a transition between hematopoietic progenitors and their differentiated descendants
Source: Development. 2021 Dec 17;148(24):dev200216. doi: 10.1242/dev.200216 (PMC8722385; doi:10.1242/dev.200216)
Supplement: Reviewer comments [file develop-148-200216_review_history.pdf]

Intermediate Progenitor cells provide a transition between hematopoietic progenitors and their differentiated descendants

Carrie M Spratford, Lauren M Goins, Fangtao Chi, Juliet R Girard, Savannah N Macias, Vivien W Ho and Utpal Banerjee

DOI: 10.1242/dev.200216

Editor: Thomas Lecuit

Review timeline

Original submission:	17 May 2021
Editorial decision:	29 July 2021
Resubmission:	20 September 2021
Accepted:	29 October 2021

Original submission

First decision letter

MS ID#: DEVELOP/2021/199599

MS TITLE: Intermediate Progenitor cells provide a transition between hematopoietic progenitors and their differentiated descendants

AUTHORS: Carrie M Spratford, Lauren M Goins, Fangtao Chi, Juliet R Girard, Savannah N Macias, Vivien W Ho, and Utpal Banerjee

I am terribly sorry and apologize for coming back to you with such a delay. This is due to one of the reports not coming back and the fact that this was not "detected" by us. I have now received the referees reports on the above manuscript, and have reached a decision. The referees' comments are appended below, or you can access them online: please go to BenchPress and click on the 'Manuscripts with Decisions' queue in the Author Area.

The overall evaluation is quite positive, though one of the reviewers expects further analysis. In light of the fact that this is a Technique and Ressource article, I believe the manuscript would be suitable for publication provided that the referees' comments can be satisfactorily addressed. Please attend to all of the reviewers' comments in your revised manuscript and detail them in your point-by-point response. If you do not agree with any of their criticisms or suggestions explain clearly why this is so.

We are aware that you may currently be unable to access the lab to undertake experimental revisions. If it would be helpful, we encourage you to contact us to discuss your revision in greater detail. Please send us a point-by-point response indicating where you are able to address concerns raised (either experimentally or by changes to the text) and where you will not be able to do so within the normal timeframe of a revision. We will then provide further guidance. Please also note that we are happy to extend revision timeframes as necessary.

© 2021. Published by The Company of Biologists under the terms of the Creative Commons Attribution License (https://creativecommons.org/licenses/by/4.0/).

First revision

Author response to reviewers' comments

Reviewer 1

… this is a terrific manuscript. Some suggestions to improve certain aspect of the manuscript are listed below as "general comments", in particular a few simple follow up experiments could greatly extend the mechanistic insight provided in the manuscript. I wouldn't classify these as essential and I would leave the decision to follow these suggestions at the discretion of the authors. However, also listed below are a number of "technical comments", mostly dealing with quantifying data to strengthen their conclusions, that need addressing in a revision.

1A. We are very grateful for the generous compliments and we have addressed these valuable suggestions, both left optional by the reviewer and the technical ones under the required category.

In Figure 1M-N, the author state that IPs were not mitotically active. However, the author based this on staining done at the wandering 3rd instar larval lymph glands. In theory it could be that IPs in mid-2nd or early-3rd (Figure 1F-G) are proliferative. To test this, the author could stain mid-2nd and early-3rd lymph glands with IPs marked by CHIZ-gal4 using anti-pH3 antibody.

1B. This is an important question and suggestion that we spent some time investigating. The simple answer continues to be that the proliferation of *CHIZ* cells is low at all stages. The percentage of *CHIZ* positive cells that are also PH3 positive in the 2nd instar is 2.2% compared with 0.6% in the 3rd. We do not feel confident reporting these statistics because the total number of cells in the 2nd instar is very low (average of 1 cell per lymph gland that is colocalized), the number of *CHIZ* positive cells increases with development, which indicates that more *CHIZ* cells are being produced from the MZ precursors rather than because they divide **(page 8)**.

We had described this as a lack of M phase in the previous manuscript. After considering the reviewer's question, this was an overreach as it is based on snapshots in development. The data that we now include accounts for these issues **(new Figure 1N, pages 8 and 13)**. We compare the phenotypes of *Aurora B- RNAi* driven by *dome* or *CHIZ* drivers. The result will be the cumulative signature of mitosis since the cells start expressing the driver (and therefore, the RNAi). AuroraB deficient cells will block when they attempt to divide (hence our reference to M phase, that we have now tempered down in the current revision. But as the **new Figure 1--figure supplement 1L** clearly shows, there is a dramatic reduction in the number of *dome*+ cells in *dome>AuroraB^{RNAi}* but no change in *CHIZ*+ cells in *CHIZ>AuroraB^{RNAi}*. We hope this addresses the reviewer's question and we thank them very much for this valuable suggestion.

In Figure 3, the authors show that over-activation of the Ras/Raf pathway resulted in reduced numbers of IPs. However, the authors could use their experimental approach to distinguish two possibilities of why IPs cell number is reduced. Namely, 1) does the Ras/Raf pathway block the entry of core progenitors into IP state or
2) does the Ras/Raf pathway facilitate cells exit out of the IP state and entry into mature blood cell state? The author could measure the number of core progenitors (cells that are only dome+), IPs (cells that are CHIZ+), and mature blood cells (cells that are only Hml+) using the genetic setup in Figure 1C upon Ras/Raf over- activation and compare that to control group to distinguish the two possibilities.

Also related to above,

In Figure 3, the author show that Ras/Raf regulates IP differentiation into hemocytes. Since hemocytes in this experiment were marked by Hml-dsred, it is unclear which differentiation trajectory (IPs to crystal cells or IPs to plasmatocytes) are specifically regulated by Ras/Raf. The author could follow the simple experimental approach using the genetic manipulations

© 2021. Published by The Company of Biologists under the terms of the Creative Commons Attribution License (https://creativecommons.org/licenses/by/4.0/).

done in IPs in Figure 3G-L (for RasV12 and RasDN) and stain for Hnt and P1 antibodies.

1C. The steps of differentiation alluded to in this reviewer's comments are fully explored in Girard *et al.*, 2021, and therefore are not repeated in this manuscript. We can confirm that the loss of Ras/Raf affects plasmatocytes while loss of Notch affects the differentiation of crystal cells. The use of a *CHIZ>pnt-RNAi* genotype helped determine that a loss of Ras/MAPK in IPs does not affect the ability of cells to differentiate into the crystal cell fate, but it does greatly hinder the trajectory into plasmatocytes. This paper is currently in revision and available as **bioRxiv #2021.02.11.430681**.

In Figure 4, the author showed that knockdown of Ser/Notch signaling specifically in IPs resulted in a reduction in the number of crystal cells. Does loss of Ser block core progenitors from transitioning into IPs which results in a lower number of IPs that can differentiate into crystal cells? The author could mark core progenitors using the genetic setup in Figure 1C (core progenitors are marked only by dome+, IPs can be marked by CHIZ>GFP, no need to have Hml+ in this case) and measure the number of two populations upon CHIZ>Ser RNAi.

1D. These are valuable comments. We have addressed them by comparing IP (*CHIZ>mGFP+*) cells in a wild type vs a *CHIZ>Ser-RNAi* background. There is no decrease in the number of *CHIZ* cells in a Ser mutant background, in fact, we observe a statistical increase in the number of IPs. The reason for the increase is unknown **(new Figure 4--figure supplement 1B; page 12)**

Technical comments:

Figure 1:

Figure 1C-E, quantification of the number of core progenitors (only dome+), IPs (CHIZ+), and mature blood cells (only Hml+) is required.

1F. We have quantitated the different fluorescently labeled populations and included these data **(new Figure 1--figure supplement 1D)**. We found that at second instar, the respective percentages of cells in the PSC, MZ, IZ, and CZ are 2, 65, 22, and 11 whereas in the third instar the corresponding percentages are 1, 28, 30, and 42. These quantifications, a representative second instar image, and text describing these data are now included in the manuscript **(new Figure 1—figure supplement 1A-D: its new Figure legend; page 25)**.

Figure 1F-H, quantification of the number of CHIZ cells in different stages is required.

1G. This is a valuable suggestion. We now provide a detailed analysis of the number of *CHIZ*+, Hnt+, and total cells **(new Figure 4A)** throughout the time course of lymph gland development. This is also addressed in **(new Figure 1--figure supplement 1D; its new Figure legend; page 25)**, where we provide the proportions of these cells at different stages.

Figure 1N, statistical analysis is missing.

1H. This statistical analysis is now provided **(new Figure 1N)**.

Figure 2:

Figure 2G is not a representative image as the quantification (Figure 2H) showed that there are roughly 50% decrease in the number of P1 cells upon CHIZ>hid or CHIS>rpr. The author should replace the image with a more representative image.

1I. We now provide an image representative of the mean number **(new Figure 2G)** of the cells in **Figure 2H**.

Figure 3:

The author showed images of cell cycle phases upon genetic manipulations in Figure 3A-E, but the result is not quantified. The author should quantify cell cycle phases in Figure 3A-E and discuss the results in text. Since the author showed that IPs contribute to mature hemocytes directly, in Figure 3G-L, it's better to do quantification of the number of Hml+ and CHIZ+ cells to understand how perturbation of the Raf/Ras disturbs the balance between IP supply and IP differentiation.

© 2021. Published by The Company of Biologists under the terms of the Creative Commons Attribution License (https://creativecommons.org/licenses/by/4.0/).

1J. Thanks, we now include quantification of the cell cycle profiles of the lymph glands **(Figure 3A-E; new Figure 3—figure supplement 1A)**. Our quantifications reveal that Ras/Raf inhibited IPs have an increased number of cells in G2, whereas activation of Ras/Raf causes more of the remaining IPs to be in S phase. As mentioned in response **1C** above, the role of Pnt, Ras/Raf are addressed in another paper (Girard et al., 2021) and therefore not repeated here.

Figure 3N, statistical analysis is missing.

1K. Thank you for pointing this out, we have now included this statistical analysis including standard deviation marked by the error bars (**Figure 3N; page 10)**.

Figure 4:
Figure 4A, data from multiple samples are required and should be incorporated into the graph showing average number of cells (GFP+ and Hnt+) and their standard deviation.

1L. We have included the standard deviation of the lymph gland samples for each timepoint as well as the quantitation of the total cells (DNA marker) for each LG **(new Figure 4A)**.

Figure 4E, the author should separate the green and magenta channels so that the readers are easier to see Notch ICD staining in individual CHIZ cells.

1M. We have now done so **(new Figure 4F)** and this improved the display.

Figure 4J, The author should quantify the number of CHIZ cells upon Ser-RNAi.

1N. This is addressed in response **1D** above.

Methods:
In method section line 413-416, the author mentioned that only the middle third of the z stack of samples were showed in images. The author should provide reasonable explanations of why the first and the last third of the lymph gland z stacks were excluded.

1O. Using the middle third as a representation is employed by multiple investigators in the field. This allows easy access to the “inside” of the lymph gland without the loss of data due to small numbers of cells in single sections, While most quantitations are for the entire stacks of the lymph gland, a maximum projection used as a display would be dominated by expression of antigens specific to the distal, CZ regions. We have updated the manuscript **(page 21)** to include this information.

Citation and references:
The author cited a paper Girard et al., 2021 a lot in their manuscript but it seems that this paper was not placed properly in the reference section.

1P. The above-mentioned paper is in the final stages of minor revisions for eLife. Similar to this *Development* manuscript, it is in the bioRx archive. The proper citations will be sorted out by the time either is officially published. This paper is available as **bioRxiv #2021.02.11.430681**. The current citation follows journal guidelines and we now include it in our reference section **(page 32)**.

Reviewer 2

The transition from progenitors to several types of differentiated blood cells has been studied in extreme details in the Drosophila hematopoietic lineage, with a major contribution from the senior author's lab. This paper addresses a different question, which is the existence of a population of transient cells that are neither progenitors nor differentiated cells and are present at the junction of the medulla and cortex of the lymph gland. These cells express markers of progenitors and markers of differentiated cells and have been defined in the past mostly through this overlap as well as their position, making it difficult to study them in isolation. These (Rubin/Janelia) lines are likely not appropriate for the hematopoietic system.
The authors can thus study these cells with this split Gal4. This allows them to make several

© 2021. Published by The Company of Biologists under the terms of the Creative Commons Attribution License (https://creativecommons.org/licenses/by/4.0/).

observations:

- Using Fly-FUCCI, they first show that these cells do not enter mitosis and indeed never express phosphorylated Histone H3: this shows that these cells are in a pre-mitotic state. However, the significance of this interesting observation is not explored.

2A. In response to this point by both reviewers, we have made several modifications to describe the mitotic status of the IPs. In particular, although PH3 incorporation is virtually missing in the third instar larvae we have changed our description to imply a relative lack of mitosis rather than our previous statement that IPs never express phosphorylated Histone3 and do not ever enter M phase. Further details are provided above in the response marked **1B**. We thank both reviewers for bringing this to our attention and the manuscript has benefitted from these changes.

- Then, they show that these cells can commit to any of the three cell types, plasmatocyte, crystal cells or Lamellocytes. Again, here, the significance of this observation is not clear.

2B. This comment is appreciated, the idea is that this IP population, although clearly different from the MZ progenitors, continues to be pluripotent for the differentiated blood cell types. This is a straightforward lineage tracing experiment, but we did not discuss the significance extensively and hope that we have now better addressed this in the discussion **(page 13)**. MZ and IPs are both multipotent. IPs but not MZ cells generate a differentiation signal for crystal cells.

- They also show that these cells need the Ras/Raf pathway to exit their Intermediate state. But what is the signal that activates Ras and what do cells do during this phase that might be required for their future differentiation?

2C. What activates Ras/Raf is unknown, and we have looked for several potential RTKs. The Agosto- Martinez laboratory has suggested a role for FGFR in the process (Dragojlovic-Munther, M., and J. A. Martinez-Agosto, 2013; Dev. Biol. 384: 313–330), unfortunately these results are not reproducible in our hands. In Girard et al., 2021 we discuss potential metabolic signals that could activate Ras. This is an ongoing investigation in our lab and is beyond the scope of this paper. Discussed in this revision **(14-15)**.

- Then, they show that Serrate expressed in these cells is necessary to induce the crystal cell fate. How is this consistent with the fact that these cells can become any of the three cell types?

2D. Thanks for the question. The IP cells are multipotent and can give rise to all hemocyte types (as per lineage trace), and Notch is expressed very widely but all neighbors of IPs do not become CCs. As in most situations in *Drosophila*, the control is at the level of the dynamics of Serrate expression and activity of RTK pathways that in this case, drives cells to a plasmatocyte fate. Dynamism of Serrate is not only over time, but often depends on environmental conditions such as the CO_2/O_2 ratio (Cho et al., 2018 Nat. Commun. 9: 2679). We have now extensively discussed the notion of dynamic Serrate expression and its role in both inducing and limiting the number of crystal cells **(pages 11-12, 14-16)**. We would like to add that although a cell bears a ligand that converts its neighbor to a particular fate, the signaling cell is free to choose amongst multiple fates depending on the signals it receives.

In conclusion, this is a nice paper that takes advantage of a new tool to focus on Intermediate Progenitors. This allows the authors to make a number of interesting observations but their significance is not addressed. At the end, we know a little more about a population of cells that were known to exist but we do not know how they contribute to the generation of the hematopoietic lineage.

2E. Thanks, the ultimate goal is to determine what role such transitional states play in hematopoiesis. It will take some time to translate these findings to a full understanding of the hematopoietic lineage. The effort described in this manuscript is an attempt to prove that such intermediary cell types exist and they have functional significance beyond what was appreciated in the past.

© 2021. Published by The Company of Biologists under the terms of the Creative Commons Attribution License (https://creativecommons.org/licenses/by/4.0/).

Reviewer 2 Comments for the Author:

- ***The last sentence of the abstract about split Gal4 in vertebrates is awkward as this is not at all the point of the paper***

  2F. We agree. We have altered this sentence from its emphasis on the possible use of split GAL4 in mammalian systems **(page 2)**.

- ***The IP cells express both dome and Pxn but the split Gal4 instead uses dome and Hemolectin.*** *This should be explained.*

  2G. This is purely for practical reasons. We now say, *Pxn* could have been used as well **(page 6)**.

Resubmission

First decision letter

MS ID#: DEVELOP/2021/200216

MS TITLE: Intermediate Progenitor cells provide a transition between hematopoietic progenitors and their differentiated descendants

AUTHORS: Carrie M Spratford, Lauren M Goins, Fangtao Chi, Juliet R Girard, Savannah N Macias, Vivien W Ho, and Utpal Banerjee
ARTICLE TYPE: Techniques and Resources Article

I apologise for the delay. I am happy to tell you that your manuscript has been accepted for publication in Development, pending our standard ethics checks. At the proof stage, please add scale bars as mentioned by reviewer 3, fix consistency of gene/protein nomenclature and reference splitGal4.

Reviewer 1

Advance summary and potential significance to field

In their manuscript, Spratford et al., demonstrated a new split-gal4 genetic tool known as CHIZ-gal4, which was applied to characterize intermediary progenitors (IPs) in detail from multiple perspectives. First, the author show that CHIZ-gal4 can mark specifically IPs in the lymph gland. The author identified that these IPs lack an MZ marker (E-cad) and several mature blood cell markers (P1 and Hnt), suggesting that the IPs are a unique cell population in the lymph gland. Second using a FUCCI tool that was designed to track cell cycle phases, the author identified that the majority of IPs stay in S and G2 phases, a smaller proportion of IPs stays in G1 phase, while no IPs are mitotically active. Third, the author demonstrated that IPs directly contribute to the mature hemocyte pool.

Specifically, they showed that ablation of IPs resulted in significant shrinkage of the cortical zone where mature hemocytes reside in. Moreover, lineage tracing experiments showed that the IPs can differentiate into all three hemocyte lineages including plasmatocytes, crystal cells, and lamellocytes. Finally, to uncover how IP population dynamics are regulated, the authors chose to manipulate multiple signaling pathways that have been implicated in controlling blood cell differentiation. Specifically, the author looked at Ras/Raf/MAPK and Notch signaling: 1) They found that over-activation of Ras/Raf pathway resulted in a reduction in the number of IPs and 2) They found that down-regulation of Ser/Notch signaling in IPs reduced the number of crystal cells. In general, this is an important and timely piece of work, the experiments in the manuscript were well-controlled, antibody stainings were convincingly validated using RNAi-mediated knockdown experiments, and the statistics/sample sizes are of high quality. This study contributes a very useful genetic tool into the fly hematopoiesis field for precise observation and manipulation of IPs. The manuscript is clear, well organized and well written.

© 2021. Published by The Company of Biologists under the terms of the Creative Commons Attribution License (https://creativecommons.org/licenses/by/4.0/).

Comments for the author

The authors have nicely addressed all the points raised by the reviewers. This is an excellent contribution to the field.

Reviewer 2

Advance summary and potential significance to field

This work represents a thorough and methodical approach to characterize a distinct population of cells that serves as an intermediate (and potential buffer) in the differentiation of three distinct blood cells in the Drosophila larva. Clearly additional questions remain (such as the source of Ras activation), but the paper provides the conceptual and technical tools to address them in the future.
Furthermore, it may provide a conceptual framework to examine similar cell types in vertebrates. While the presence of intermediate cell fates has been previously demonstrated in different systems by single-cell RNAseq, the strength of this paper is the ability to carry out the entire analysis in the intact organ, in the context of the whole organism.

Comments for the author

It seems to me that the issues raised by the two reviewers have been properly addressed.

Reviewer 3

Advance summary and potential significance to field

Intermediate progenitor (IP) cells in the lymph gland are characterized by the transient and overlapping expression of domeless (dome, early progenitors) and hemolectin (hml) before they differentiate into mature blood cells including plasmatocytes, crystal cells, and lamellocytes. Using the enhancers from the dome and hml genes in a split Gal4 system together with a short-lived GFP, Spratford et al. describe a novel driver line that faithfully identifies the intermediate progenitor cells in the lymph gland during Drosophila development. Using this new split Gal4 line, the authors characterize the IP cells in detail and demonstrate that IPs contribute to all mature hemocyte populations in a Ras/Raf and Notch-dependent manner. The authors have performed most of the necessary experiments to support their conclusions, and the data are generally beautiful. Overall, this work adds extensive detail to our understanding of lymph gland IPs, and presents an effective new tool for further studies.

Comments for the author

We have the following specific comments:
1. The gene and protein nomenclature should be more consistent. For example, in domeless versus Hemolectin, Peroxidasin etc., one starts with lowercase italics while the other starts with uppercase straight.
2. The "split GAL4" system needs to be briefly explained and appropriately referenced. It is nicely diagrammed in Fig 1B, but adding a few sentences about how it works in the main text would be helpful.
3. None of the images have scale bars. Scale bars are necessary to compare the effects of the genetic manipulations. Figure panels 2L –N are difficult to contextualize. The absence of sale bars makes it even more difficult to understand. Please add scale bars to all images. Additionally, lower magnification images, as in Figure 2, C-J, would be helpful.
4. Adding a proper stage-specific quantification of the cell lineages in Figure 2L-N would be informative, and helpful in providing a complete picture of the IP developmental hemocyte maturation process.
5. It is difficult to interpret the IF images in Figure 3M –Q. Additional zoomed-out images with nuclear counterstain would make the panels easier to assess. The graph in Figure 3L should include quantification of the Yan and dpERK in CHIZ+ and Lz+ cells.

© 2021. Published by The Company of Biologists under the terms of the Creative Commons Attribution License (https://creativecommons.org/licenses/by/4.0/).

6. The title looks to be too generic and does not inform the reader of the novel findings in the manuscript. Please modify it to reflect the authors' work specifically.
7. The discussion section, especially lines 296-316, reads like an unnecessary extrapolation. The authors can either eliminate this part or summarize it in fewer sentences.

© 2021. Published by The Company of Biologists under the terms of the Creative Commons Attribution License (https://creativecommons.org/licenses/by/4.0/).
